# *Plasmodium vivax* Malaria Relapses after Primaquine Prophylaxis

**DOI:** 10.3201/eid1211.060613

**Published:** 2006-11

**Authors:** Pavani Reddy, John P. Flaherty

**Affiliations:** * Northwestern University Feinberg School of Medicine, Chicago, Illinois, USA

**Keywords:** Plasmodium vivax, primaquine, failure, relapse, resistance, letter

To the Editor: Standard treatment of patients with Plasmodium vivax malaria includes chloroquine, followed by primaquine terminal prophylaxis. Reports of true primaquine failure and subsequent P. vivax relapse are unusual; most suspected cases can be ascribed to poor patient adherence, recrudescence of a chloroquine-resistant strain, or P. vivax reinfection. We report a case of P. vivax malaria relapse after therapy with quinine, doxycycline, and primaquine, and again after treatment with chloroquine and primaquine. P. vivax relapses after primaquine treatment are exceedingly rare in travelers to South America and are a serious therapeutic challenge. Our patient was subsequently treated with weekly, single-dose chloroquine without recurrence of symptoms.

A 77-year-old man had fever and chills 2 weeks after returning from Brazil. These symptoms were accompanied by sweating, fatigue, and a mild, productive cough. Review of systems was notable for dark, concentrated urine and a 10-lb weight loss. The patient's 25-day journey included Salvador, Manaus, and a 2-day stay in the Amazon River basin. He did not take malaria prophylaxis during his trip.

On physical examination, the patient was afebrile with blood pressure of 90/53 mm Hg. Cardiovascular, pulmonary, and abdominal examination results were unremarkable. Several petechiae were noted on both lower extremities. Laboratory tests showed the following: leukocyte count 6,300 cells/μL, hemoglobin level 13.7 g/dL, platelet count 40,000 cells/μL, serum creatinine level 1.2 mg/dL, serum alanine aminotransferase level 63 IU/L, and serum aspartate aminotransferase level 56 IU/L. Thick and thin peripheral blood smears revealed P. vivax with a parasitemia level of 0.67%. Although the existence of chloroquine-resistant P. vivax in Brazil is debatable, the patient was conservatively treated with quinine, 650 mg, 3×/day and doxycycline, 100 mg, 2×/day for 7 days, followed by primaquine terminal prophylaxis, 30 mg/day for 30 days with complete resolution of symptoms.

In the absence of travel abroad, the patient experienced similar symptoms 5 months later. On the basis of thick and thin peripheral blood smear examination, a relapse of P. vivax malaria was diagnosed. He was given chloroquine, 2.5 g over 3 days, followed by primaquine, 30 mg/day for 30 days. Again, the patient's symptoms resolved.

Four months after treatment (9 months after the initial episode), the patient experienced the abrupt onset of fever, chills, and dark urine. He had a leukocyte count of 5,900 cells/μL, a hemoglobin level of 14.0 g/dL, and a platelet count of 117,000 cells/μL. Repeat thick and thin blood smears showed P. vivax with a parasitemia level of 0.993%. Therapy with chloroquine was initiated (2.5 g over 3 days), and symptoms resolved. Repeat blood smears 4 days later were negative for P. vivax. In lieu of yet another course of terminal prophylaxis with primaquine, the patient was given chloroquine, 300 mg/week for 4 months; he has been asymptomatic for an additional 2 months.

Even before Food and Drug Administration approval of primaquine in 1951, primaquine failure was documented in experimental cases of the Chesson (tropical) P. vivax strain (*1*). Additional reports soon followed, citing dosing differences as the likely reason for P. vivax relapse. Baird and Hoffman summarized cases of primaquine failure over nearly 3 decades, noting that 26 (25%) of 103 patients given primaquine, 15 mg/day for 14 days, relapsed, while infection returned in only 1 (3.9%) of 26 patients given 22.5–30 mg/day (*2*). Among 50 patients treated for P. vivax malaria in Brazil, total primaquine dose per patient was the only variable in relapse; 7 relapses occurred in patients who received 2.76 mg/kg, while those who received 3.35 mg/kg remained free of infection (*3*). As a consequence, patients weighing >70–80 kg should receive 0.5 mg/kg/day (*2*).

The issue of primaquine resistance in P. vivax remains unresolved for several reasons. First, the organism cannot be propagated in vitro, and injection of P. vivax into nonhuman primates is required for analysis (*4*). Second, the pharmacokinetics of primaquine are poorly understood. Despite standard dose administration, 1 study suggested substantial interethnic differences in peak plasma concentrations of primaquine and its major metabolite, carboxyprimaquine (*5*). Finally, confounding factors such as drug dosing and patient compliance have complicated most failure reports.

Our patient initially received quinine and doxycycline, which excluded a chloroquine-resistant infection. In addition, he completed a primaquine regimen of 10.8 mg/kg, which is twice the current recommended dose. In the absence of reexposure, the patient had a relapse 5 months later. His condition was treated with chloroquine and again with high-dose primaquine. He reported strict adherence to the treatment regimen, citing the fastidious use of a weekly pill box as evidence. Despite these measures, another relapse occurred 4 months later. This patient's course suggests P. vivax primaquine failure and possible resistance. When high-dose regimens of primaquine (total 5–6 mg/kg) fail, suppressive doses of chloroquine, 300 mg/week for several months to years may be considered. Our patient received chloroquine therapy, 300 mg/week for 4 months without evidence of recurrence.
